# ATF4-dependent and independent mitokine secretion from OPA1 deficient skeletal muscle in mice is sexually dimorphic

**DOI:** 10.3389/fendo.2024.1325286

**Published:** 2024-09-24

**Authors:** Jennifer Streeter, Luis Persaud, Jason Gao, Deeraj Manika, Will Fairman, Luis Miguel García-Peña, Alex Marti, Chethan Manika, Shreya Gaddi, Brandon Schickling, Renata O. Pereira, E. Dale Abel

**Affiliations:** ^1^ Department of Internal Medicine, Carver College of Medicine, University of Iowa, Iowa City, IA, United States; ^2^ Department of Obstetrics and Gynecology, Carver College of Medicine, University of Iowa, Iowa City, IA, United States; ^3^ Department of Medicine, David Geffen School of Medicine, University of California Los Angeles, Los Angeles, CA, United States

**Keywords:** mitochondria, integrated stress response, insulin resistance, obesity, FGF21

## Abstract

**Introduction:**

Reducing Optic Atrophy 1 (OPA1) expression in skeletal muscle in male mice induces Activation Transcription Factor 4 (ATF4) and the integrated stress response (ISR). Additionally, skeletal muscle secretion of Fibroblast Growth Factor 21 (FGF21) is increased, which mediates metabolic adaptations including resistance to diet-induced obesity (DIO) and glucose intolerance in these mice. Although FGF21 induction in this model can be reversed with pharmacological attenuation of ER stress, it remains to be determined if ATF4 is responsible for FGF21 induction and its metabolic benefits in this model.

**Methods:**

We generated mice with homozygous floxed *Opa1* and *Atf4* alleles and a tamoxifen-inducible Cre transgene controlled by the human skeletal actin promoter to enable simultaneous depletion of OPA1 and ATF4 in skeletal muscle (mAO DKO). Mice were fed high fat (HFD) or control diet and evaluated for ISR activation, body mass, fat mass, glucose tolerance, insulin tolerance and circulating concentrations of FGF21 and growth differentiation factor 15 (GDF15).

**Results:**

In mAO DKO mice, ATF4 induction is absent. Other indices of ISR activation, including XBP1s, ATF6, and CHOP were induced in mAO DKO males, but not in mOPA1 or mAO DKO females. Resistance to diet-induced obesity was not reversed in mAO DKO mice of both sexes. Circulating FGF21 and GDF15 illustrated sexually dimorphic patterns. Loss of OPA1 in skeletal muscle increases circulating FGF21 in mOPA1 males, but not in mOPA1 females. Additional loss of ATF4 decreased circulating FGF21 in mAO DKO male mice, but increased circulating FGF21 in female mAO DKO mice. Conversely, circulating GDF15 was increased in mAO DKO males and mOPA1 females, but not in mAO DKO females.

**Conclusion:**

Sex differences exist in the transcriptional outputs of the ISR following OPA deletion in skeletal muscle. Deletion of ATF4 in male and female OPA1 KO mice does not reverse the resistance to DIO. Induction of circulating FGF21 is ATF4 dependent in males, whereas induction of circulating GDF15 is ATF4 dependent in females. Elevated GDF15 in males and FGF21 in females could reflect activation by other transcriptional outputs of the ISR, that maintain mitokine-dependent metabolic protection in an ATF4-independent manner.

## Graphical Abstract

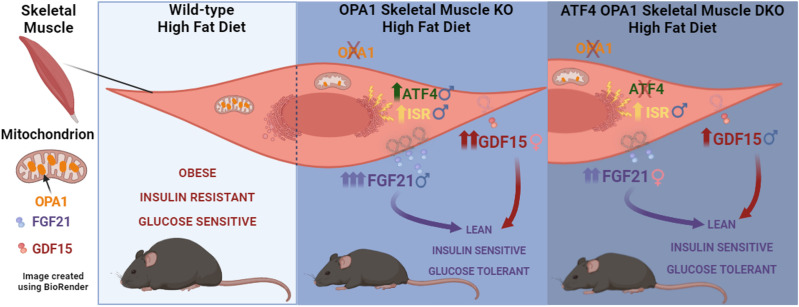

## Introduction

Activating Transcription Factor 4 (ATF4) is a key transcription factor induced in response to endoplasmic reticulum (ER) stress and mitochondrial stress. *Atf4* expression is tightly regulated at the transcriptional level and requires multiple steps for activation. ER stress leads to PERK (Protein kinase R-like ER kinase) activation via oligomerization and auto-phosphorylation. Activated PERK phosphorylates eukaryotic transcription initiation factor 2α (eIF2α), thereby inactivating eIF2α and inhibiting its mRNA translation. Downregulation of eIF2a causes the preferential translation of ATF4, which drives the transcription of the coordinated gene expression program known as the integrated stress response (ISR) which regulates apoptosis, ER stress negative feedback, lipid synthesis, ER-associated protein degradation, and expression of ER chaperones (1, 2).

FGF21 is an important hormone that serves as a regulator of whole-body energy homeostasis in response to various stressors such as starvation or nutrient excess (3, 4). It is predominantly expressed in the liver, but is also expressed in adipose tissue, pancreas, and can be induced in brain, cardiac tissue, and skeletal muscle (5–12). Studies have demonstrated that ATF4 is a key regulator of *Fgf21* expression in various tissues (11, 13, 14). The 5’ regulatory region of the *Fgf21* gene harbors three ATF4-binding sequences known as amino acid response elements AARE1, AARE2, and AARE3 (15, 16). In hepatocytes, mutation of these binding sites abrogates *Fgf21* promoter activity induced by *Atf4* overexpression (3, 17–19). Several *in vitro* studies suggest that ATF4 may also regulate *Fgf21* expression in skeletal muscle. Kim et al. showed that overexpression of ATF4 in C2C12 myotubes increases *Fgf21* expression, while knockdown of ATF4 by siRNA inhibits rotenone-induced *Fgf21* expression. This study also demonstrated that mutation of ATF4 response elements in the *Fgf21* promoter reduces reporter activity of the *Fgf21* promoter in C2C12 myotubes. In several *in vivo* studies, inhibition of *Atf4* expression in skeletal muscle significantly reduces serum FGF21 levels (9, 20, 21). Direct binding of ATF4 to the FGF21 promotor in skeletal muscle *in vivo* has not been demonstrated but has been observed in C2C12 cells *in vitro* and human aortic vascular smooth muscle cells *in vitro* by chromatin precipitation (14, 22). Taken together, these data suggest that ATF4 plays an important role in the control of FGF21 expression and secretion in skeletal muscle.

*Atf4* and *Fgf21* expression are strongly induced following reduction of Optic Atrophy 1 (OPA1) in skeletal muscle in mice (mOPA1 KO) (9). OPA1 is an inner mitochondrial membrane protein that regulates mitochondrial fusion, assembly of respiratory chain supercomplexes, cristae organization and sequestration of cytochrome c (23). Deletion of OPA1 leads to abnormal mitochondrial morphology characterized by a significant decrease in cristae number and disorganization of remaining cristae (24). Pereira et al. demonstrated that *Opa1* deletion in skeletal muscle induces the ER stress/unfolded protein response (UPR) or ISR, characterized by increased expression of ATF4, CHOP (CCAAT enhancer binding protein homologous protein), XBP1s (X-box binding protein 1 spliced), and BiP (an ER chaperone protein also known as GRP78 and HSPA5) (9). Additionally, mOPA1 KO mice exhibit increased *Fgf21* expression in skeletal muscle in concert with increased circulating FGF21. In this model, FGF21 acts as a mitokine, i.e., a signaling molecule that communicates mitochondrial stress systemically. These mice are protected from high-fat-diet (HFD)-induced obesity (DIO), and insulin resistance relative to wildtype (WT) littermates. A double KO (DKO) mouse model deficient in OPA1 and FGF21 in skeletal muscle (mOPA1 FGF21 DKO) lacks these favorable metabolic phenotypes thereby confirming that muscle derived FGF21 is a critical mediator of protection from DIO and insulin resistance in this model.

Although ATF4 is known to be critical for *Fgf21* expression in various models and tissues, *Fgf21* can be regulated by multiple transcription factors (3, 4, 13, 25). It is currently unknown if ATF4 is necessary for *Fgf21* induction or the metabolically favorable phenotype in the mOPA1 KO model. This study was designed to determine the extent to which ATF4 regulates *Fgf21* in skeletal muscle in response to OPA1 downregulation and whether ATF4 is necessary for the favorable metabolic phenotype reported in the mOPA1 KO mouse. Additionally, there are known sex-dependent differences in the regulation of weight, obesity, and insulin resistance. Therefore, this study additionally sought to explore whether ATF4 regulates FGF21 release and the attendant metabolic adaptations in a sex-dependent manner (26).

## Materials and methods

### Animal work

Animal work was performed in accordance with protocols approved by the University of Iowa Animal Care and Use Committee (IACUC). Unless otherwise stated, all experiments were performed in mice on a C57Bl/6J background or that were backcrossed into the C57Bl/6J background for more than five generations. *OPA1^fl/fl^* mice were generated as previously described (30). *Atf4 ^fl/fl^* mice were generated as previously described (31). Tamoxifen-inducible *HSA-CreER_T2_* mice were a kind gift from Dr. Pierre Chambon, University of Strasbourg. Inducible skeletal muscle *Atf4* and *Opa1* double knock out mice were generated by crossing *Opa1^fl/fl^* mice carrying the inducible *HSA-CreER_T2_* with *Atf4^fl/fl^* mice to generate *HSA-CreER_T2_ ATF4^fl/fl^ OPA1^fl/fl^* mice (mAO DKO). Recombination in skeletal muscle was induced in 6-week-old mice via intraperitoneal injections of tamoxifen (Sigma, St. Louis, MO, USA, T5648; 20 mg/kg for males, 10 mg/kg for females) for 5 days. Mice were kept on regular chow diet until four weeks post injection (2920X Harlan Teklad, Indianapolis, IN, USA), then divided into a low-fat control diet group (CD; 10% Kcal from fat—Research Diets, New Brunswick, NJ, USA, D12450J) or a high-fat diet group (HFD; 60% Kcal from fat—Research Diets D12492) and were kept on these respective diets for 12 -22 weeks. All animal work was done in mice housed at room temperature. All serum and skeletal muscle samples were collected between 8 am and noon.

### GTT, ITT, FGF21/GDF15 ELISA and body composition by NMR

Glucose tolerance tests (GTT) were performed after a 4-h fast, and mice were administered glucose (2 g/kg body weight), as described (32). Insulin tolerance tests (ITT) were performed after a 4-h fast by injecting insulin (0.75 U/kg body weight; Humulin, Eli Lilly, Indianapolis, IN, USA). Blood glucose was determined using a glucometer (Glucometer Elite; Bayer, Tarrytown, NY, USA). Dosages were based on body weight, prepared in sterile 0.9% saline, and administered intraperitoneally. FGF21 and GDF15 serum levels were measured using commercially available kits according to the manufacturers’ directions (BioVendor ELISA kit, Asheville, NC, USA, Quantikine ELISA Mouse/Rat GDF-15, Minneapolis, MN, USA). Whole body composition was measured using the Bruker Minispec NF-90 instrument (Bruker, Billerica, MA, USA).

### Electron microscopy

Soleus muscle was excised, fixed in Karnovsky’s buffer, and processed as previously described at the University of Iowa Microscopy Core Facility (9).

### RNA extraction and qPCR

Total RNA was extracted from tissues with and purified using the mirVana RNA isolation kit (Thermo Fisher Scientific, Waltham, MA, USA) or Trizol (Thermo Fisher) extraction and concentration was determined by measuring the absorbance at 260 and 280 nm using a spectrophotometer to establish RNA concentrations and integrity (NanoDrop 1000, NanoDrop products, Wilmington, DE, USA). Total RNA was reverse transcribed using the Maxima H Minus cDNA Synthesis Master Mix with dsDNase (Thermo Fisher Scientific) followed by qPCR reactions using power SYBR Green PCR Master Mix (Life Technologies, Carlsbad, CA) (33). A QuantStudio 5 Real Time PCR System was used. A PCR reaction volume of 10µL, containing 6 µL master mix, 2 µL 4µM forward + reverse primer mix (1µM of each primer) and 2 µL cDNA sample, was thermally cycled with the following program: 95°C for 10 minutes, followed by 40 cycles of 95°C for 15s, and 60°C for 60s. A melt curve was generated by holding at 95°C for 15s, 60°C for 60s and increasing temperature at a rate of.075°C/s to 95°C, while measuring fluorescence. All temperature changes take place at 1.6°C/s unless otherwise specified. Data was quality checked and exported from QuantStudio Design & Analysis v2.8.0. Cq values were determined through QuantStudio implementation of baseline-threshold method with a threshold of 0.2 and automatic baseline determination. Data was normalized in Excel using efficiency-corrected relative quantification with a single reference gene (36B4). PCR efficiencies for each target were estimated through the generation of 12-point standard curves using 2-fold serial dilutions of pooled cDNA from all sexes and genotypes. Data were normalized to 36B4, which was minimally affected by sex or genotype. Primers used are shown in **Table 1**.

**Table 1:** qPCR primer sequences.

Gene	Forward	Reverse
36B4	ATCCCTGACGCACCGCC GTGA	TGCATCTGCTTGGAGCC CACGTT
FGF21	TGACGACCAAGACACT GAAGC	TTTGAGCTCCAGGAG ACTTTCTG
GDF15	CCGAGAGGACTCGA ACTCAG	ACCCCAATCTCA CCTCTGGA
XBP1T	ACACGTTTGGGAATG GACAC	CCATGGGAAGAT GTTCTGGG
XBP1S	GAGTCCGCAGC AGGTG	GTGTCAGAGTC CATGGGA
BIP	TGTGTGAGACCAGAA CCGTC	TAGGTGGTCCCCA AGTCGAT
CHOP	CTGCCTTTCAC CTTGGAGAC	CGTTTCCTGGGGAT GAGATA
ATF4	ATGGCCGGCTA TGGATGAT	CGAAGTCAAACTCTT TCAGATCCAATT
OPA1	TTCGGAGACGGAC TGACTAC	TTCCTGCTCCGA CAATGACC
ATF6	TGGGCAGGACTAT GAAGTAATG	CAACGACTCAGG GATGGTGCTG

### Western blot analysis

Immunoblotting analysis was performed using 50 mg of frozen tissue homogenized in 400 ul of lysis buffer comprised of 150 mmol NaCl, 10% glycerol, 1% Triton X-100, 100 mmol sodium fluoride, 50 mmol HEPES, 10 mmol sodium pyrophosphate, 1.5 mmol MgCl_2_, 1 mmol EGTA, and 100 μmol/l of sodium vanadate. HALT protease/phosphatase inhibitors (Thermo Fisher Scientific) were applied to the lysis buffers before use and all tissues were homogenized in a TissueLyser II (Qiagen Inc., Germantown, MD, USA). SDS-PAGE electrophoresis using NativePAGE 3-12% Bis-Tris gels (Invitrogen, Thermo Fisher Scientific) and NuPAGE MOPS SDS running buffer (Invitrogen, Thermo Fisher Scientific) was utilized for lysate separation and proteins were transferred to nitrocellulose membranes (BIO RAD Corp 1620115, Hercules, CA, USA). Membranes were incubated in primary and secondary antibodies for 1 hour at room temperature.

### Antibodies

Primary Antibodies: OPA1 (1:1,000, BD Biosciences, San Jose, CA, USA, #612606), GAPDH (1:2000, Santa Cruz Biotechnology #SC47724, Dallas, TX, USA), Secondary antibodies: IRDye 800CW anti-mouse (1:10,000, LI-COR, Lincoln, NE, USA, #925-32212) and IRDye 680CW (1:10,000, Invitrogen #A27042). Fluorescence was quantified using the LiCor Odyssey CLx imager (Lincoln, NE, USA).

### Geo dataset analysis

Analysis of GSE35681 ChIP-seq and RNA-seq was performed in Galaxy (27). ChIP-seq reads were mapped with bowtie (https://bowtie-bio.sourceforge.net/index.shtml). The SAM (Sequence Alignment Map) file was converted to BAM (Binary Alignment Map)and then MACs (Model-based Analysis of ChIP-Seq) was used for peak calling (28). Tracks for the peaks were visualized in the Integrated Genome Browser. RNA-seq reads were mapped with TopHat and visualized in the Integrated Genome Brower. Differential gene expression was determined using DeSeq2.

### Data analysis

Normality and lognormality of data was tested through D’Agostino & Pearson, Anderson-Darling, Shapiro-Wilk, and Kolmogorov-Smirnov tests with significance levels of 0.05. For each gene of interest, data are considered non-normally distributed if normality test results were ambiguous or positive for lognormality. All data are reported as arithmetic means ± SEM. Student’s t-test was performed for comparisons of two groups with Welch’s correction when appropriate. When more than three groups were compared, descriptive statistics and normality/lognormality tests were used to determine appropriate usage of either Brown-Forsythe and Welch ANOVA with Dunnett’s T3 multiple comparisons test or Kruskall-Wallis with Dunn multiple comparisons test. ROUT method was used to identify and remove outliers, Q=10%. A probability value of p < 0.05 was considered significantly different. Statistical calculations were implemented through GraphPad Prism software (La Jolla, CA, USA).

## Results

### ATF4 binds the FGF21 promoter and induces FGF21 expression in WT MEFs, but not in ATF4 KO MEFs

To explore the question of whether ATF4 regulates FGF21 under increased ER stress conditions, as observed in mOPA1 KO mice, we conducted GEO DataSet RNA-seq and ChIP-seq analysis of WT and *Atf4* KO mouse embryonic fibroblasts (MEFs) treated with the ER-stress-inducer tunicamycin (29). RNA-seq showed that tunicamycin treatment induces expression of ER stress markers ATF4, XBP1, ATF6, BiP, and CHOP (**Supplementary Figure 1A**). Expression analysis reveals that FGF21 expression occurs in WT MEFs, but not in *Atf4* KO MEFs (**Supplementary Figure 1B**). ChIP-seq revealed that ATF4 binds directly to the promoter of *Fgf21* in WT MEFS, but not in *Atf4* KO MEFs (**Supplementary Figure 1C**). These results suggest that ATF4 directly binds to the promoter of *Fgf21* and regulates FGF21 expression in response to tunicamycin-induced ER stress in MEFs.

### Generation of mouse model of inducible ATF4 and OPA1 knock out in skeletal muscle

**Figure 1:**
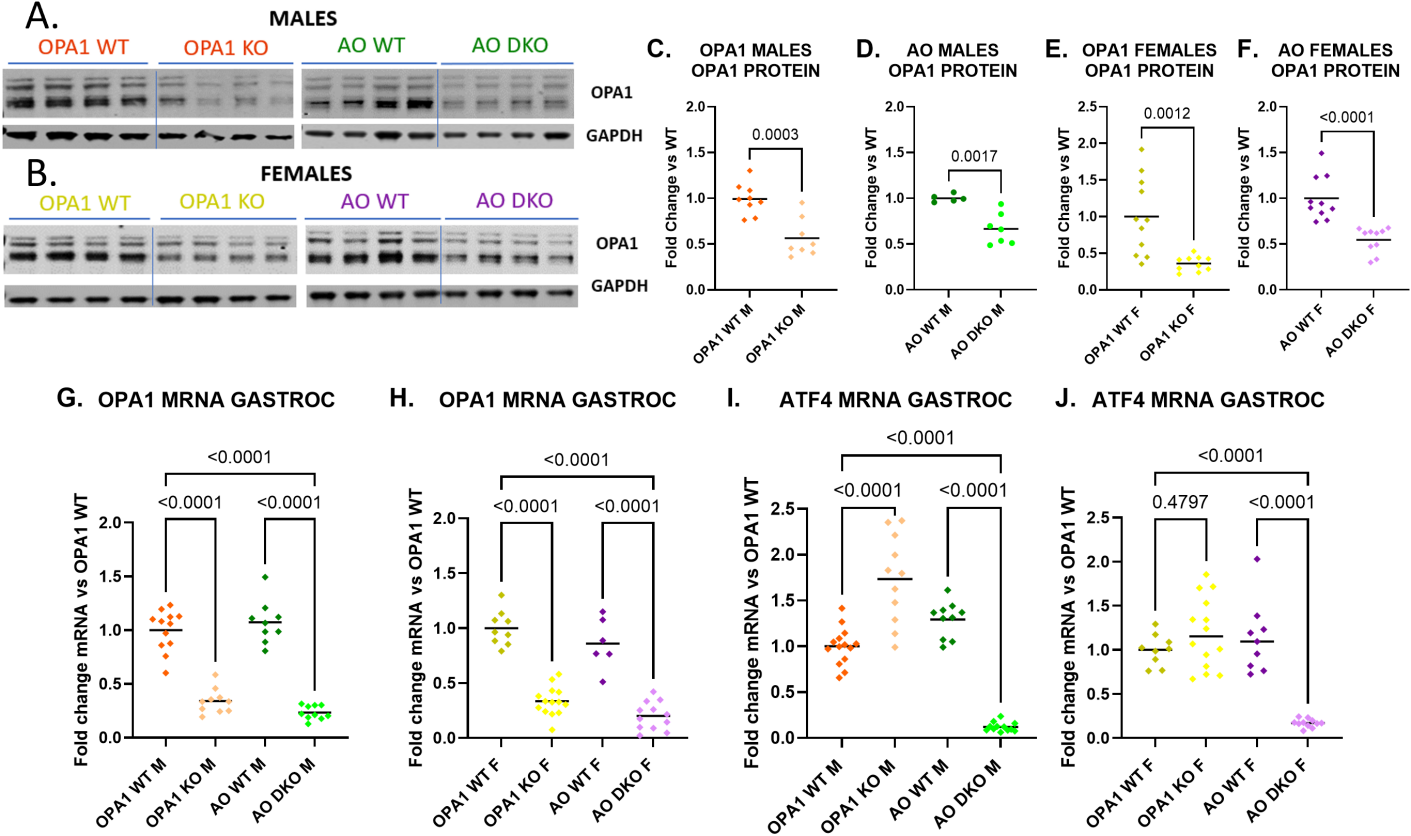
Quantification of OPA1 and ATF4 Expression in mOPA1 KO and mAO DKO mice. WT mOPA1 males (dark orange), mOPA1 KO males (light orange), WT mAO males (dark green), mAO DKO males (light green), WT MOPA1 females (dark yellow), mOPAI KO females (light yellow), WT MAO females (dark purple), and mAO DKO females (light purple). Representative immunoblots of OPA1 protein in gastrocnemius (GASTROC) muscle from 22-week-old male **(A)** and female **(B)** WT, mOPA1 knock out (KO), and mOPA1/mATF4 double KO (DKO) mice and densitometric analysis of OPA1 protein normalized by GAPDH in OPA1 males (**C**, n=5-8), AO males (**D**, n= 5-7), OPA1 females (**E**, n=11), and AO females (**F**, n=10-11). OPA1 mRNA quantification in GASTROC muscle from 22-week old males (**G**, n=9-12) and females (**H**, n=6-14), fold change vs OPA1 WT. ATF4 mRNA quantification in gastrocnemius muscle from 22-week old males (**I**, n=10-14) and females (**J**, n=9-14), fold change vs OPA1 WT. OPA1 (mOPA1 mice), AO (mATF4 OPA1 DKO mice), WT (wildtype), KO (knock out), DKO (double knockout), F (female), M (male). ROUT method was used to identify and remove outliers, Q=10%. Created using BioRender.com.

The mOPA1 KO mouse has a favorable metabolic phenotype characterized by protection from DIO and insulin resistance. Double knockout of *Opa1* and *Fgf21* in skeletal muscle (mOPA1 FGF21 DKO) confirmed that FGF21 is essential for this favorable metabolic phenotype in male mice (9). Since ATF4 is induced in mOPA1 KO males and ATF4 can regulate FGF21 expression, we sought to test the hypothesis that ATF4 is required for FGF21 expression and protection from insulin resistance and DIO in mOPA1 KO mice. Thus, we generated skeletal muscle-specific *Atf4-Opa1* double KO mice (mAO DKO) that are homozygous for both *Atf4* and *Opa1* floxed alleles, and also express a tamoxifen-inducible Cre transgene (*Cre-ER^T2^*) under the control of the human skeletal-actin (HSA) promoter (30). Excision of the floxed target genes was induced by five consecutive days of weight-based administration of tamoxifen at six weeks of age. Skeletal-muscle specific knockdown of OPA1 was verified by qPCR and Western blots (WBs), revealing significantly reduced OPA1 transcript and protein levels in mOPA1 KOs and mAO DKO males and females relative to their respective WT littermates in gastrocnemius skeletal muscle harvested at 22 weeks of age (**Figures 1A–H**). ATF4 did not have a reliable signal on WB, therefore knockdown of ATF4 was confirmed by qPCR. (**Figures 1I, J**). As previously observed in mOPA1 KO mice, qPCR demonstrated significant increase in ATF4 in mOPA1 KO males compared to WT males (**Figure 1I**). In contrast, ATF4 was not increased in mOPA1 KO females (**Figure 1J**). ATF4 was significantly decreased in gastrocnemius skeletal muscle in mAO DKO males and females compared to WT males and females, respectively (**Figures 1I, J**). In liver, there was no difference in OPA1 protein or mRNA levels (**Supplementary Figures 2A–E**), or ATF4 mRNA level (**Supplementary Figures 2F, G**) in mOPA1 KO or mAO DKO males or females relative to their WT counterparts, indicating skeletal muscle-specific deletion of these floxed alleles. Previously, it was demonstrated that mitochondria in mOPA1 KO males are characterized by loss of cristae content and abnormal vacuolated ultrastructure (9). Therefore, we assessed mitochondrial morphology in mOPA1 KO and mAO DKO males and females compared to controls and found that mOPA1 KO females and mAO DKO males and females demonstrate dysmorphic, sparse, and vacuolated cristae similar to previously reported changes in mOPA1 KO males, which was not further altered by loss of ATF4 (**Supplementary Figure 3**).

### Sexual dimorphism in ISR in male versus female mAO DKO mice

**Figure 2:**
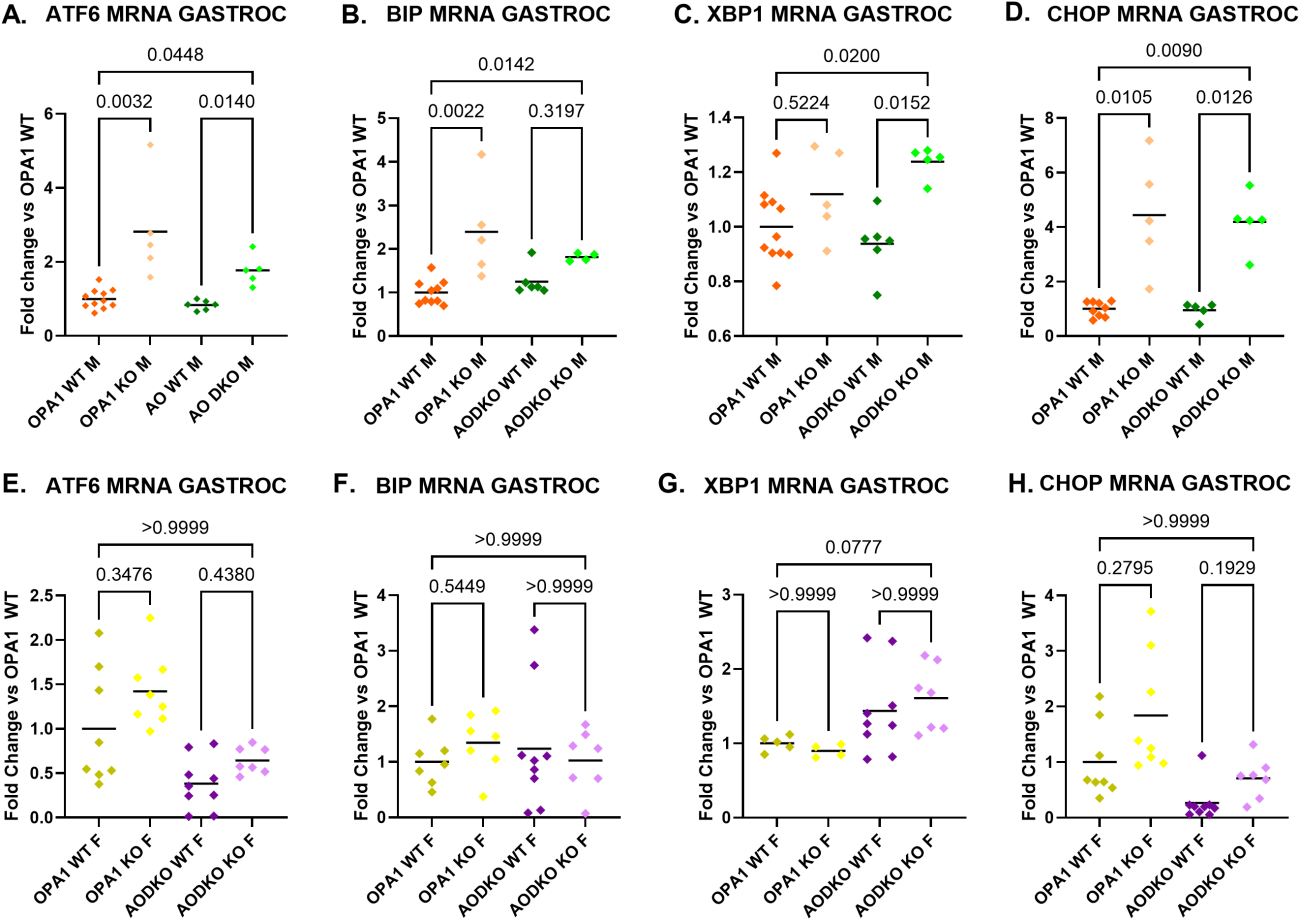
ER Stress Markers mRNA Expression: mRNA quantification in gastrocnemius (gastroc) muscle from 22-week old mOPA1 males and mAO males **(A-D)**, mOPA1 females and mAO females **(E-H)** of XBP1 S/T (spliced/total, **C** and **G**), ATF6 **(A, E)**, BiP **(B, F)**, and CHOP **(D, H)**, fold change vs OPA1 WT (n=4-11). Created using BioRender.com.

Transcript levels of ISR inducers ATF6, BiP and CHOP, but not XBP1s, were increased in mOPA1 KO males (**Figures 2A–D**). In mAO DKO males, ATF6, CHOP, and XBP1s were increased compared to WT males, indicating that activation of the ISR in mOPA1 KO male mice is ATF4-independent (**Figures 2A–D**). Conversely, the induction of BiP in mOPA1 KO males, but not mAO DKO males suggest that BiP induction is ATF4 dependent in this model. Unlike males, mOPA1 KO and mAO DKO females did not have increases in ATF6, BiP and CHOP, nor XBP1s, demonstrating sexual dimorphism in the ISR to OPA1 depletion in skeletal muscle (**Figures 2E–H**).

### ATF4 deletion does not prevent OPA1 KO-induced resistance to weight gain on HFD

**Figure 3:**
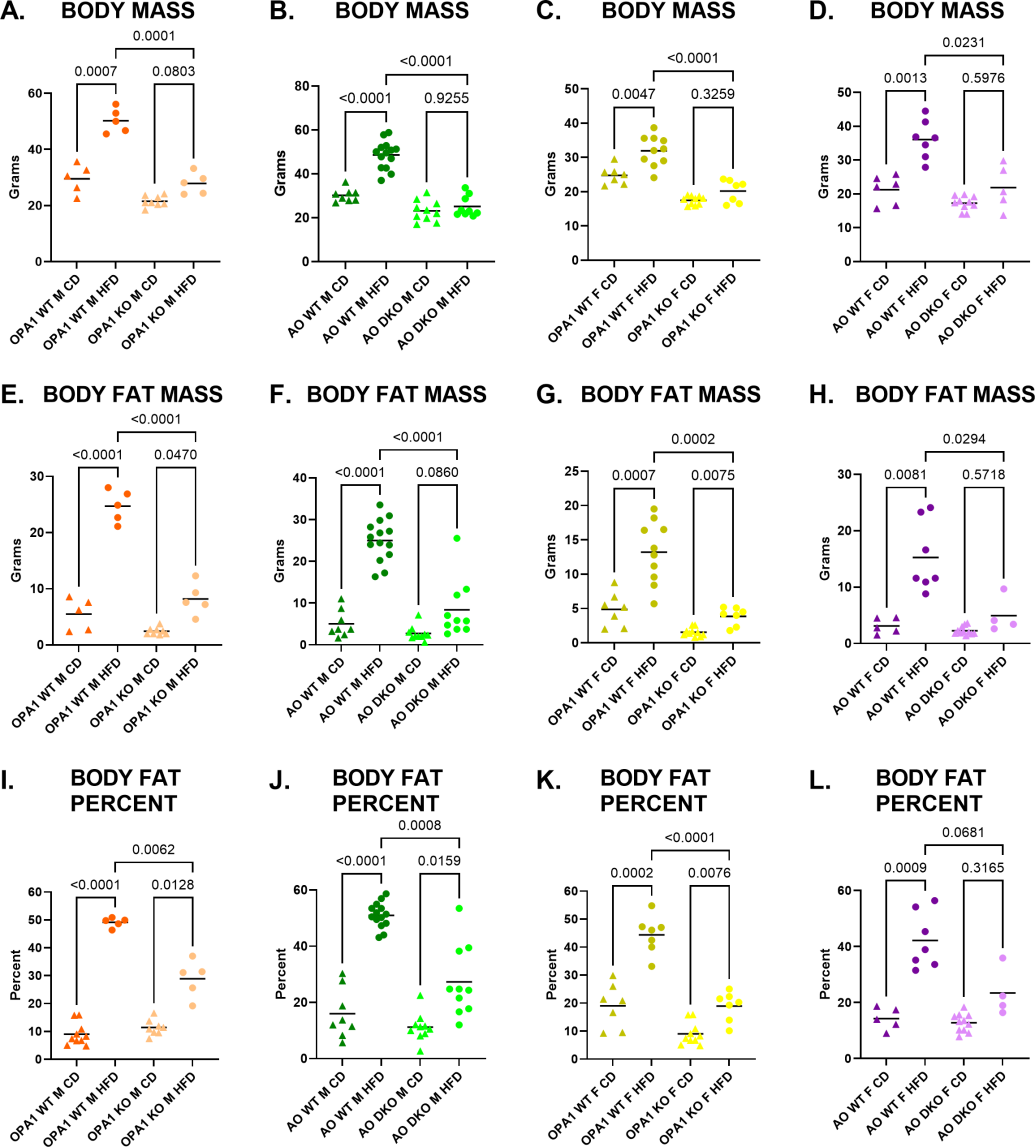
Body Mass and Fat Mass. Body mass **(A–D)**, body fat mass **(E–H)** and body fat percent **(I–L)** after 10 weeks of high fat diet (HFD, circles) or control diet (CD, triangles). P values determined through Brown-Forsythe and Welch one-way ANOVA with Dunnett’s T3 multiple comparisons test. Comparison pairs shown here: mOPA1 or mATF4 OPA1 (AO) wildtype (WT) males (M) or females (F) on CD vs HFD, WT on HFD vs mOPA1 knockout (KO) or m ATF4 OPA1 double KO (AO DKO) on HFD, and OPA1 KO or AO DKO on CD vs HFD. Created using BioRender.com.

As previously reported, mOPA1 KO males are protected from HFD-induced obesity (DIO) relative to WT males (**Figures 3A, E, I**). mOPA1 KO females are also protected from DIO compared to WT females (**Figures 3C, G, K**). Since FGF21 is necessary for protection against DIO, we hypothesized that if ATF4 was the major regulator of FGF21, then ATF4-deficient mice (mAO DKO) would not be protected from DIO. However, mAO DKO males and females remain protected from DIO relative to WTs, suggesting that ATF4 is not the sole regulator of the metabolic adaptations observed in mOPA1 KOs (**Figures 3B, D, F, H, J, L**).

### ATF4 deletion does not prevent OPA1 KO-induced muscle atrophy

**Figure 4:**
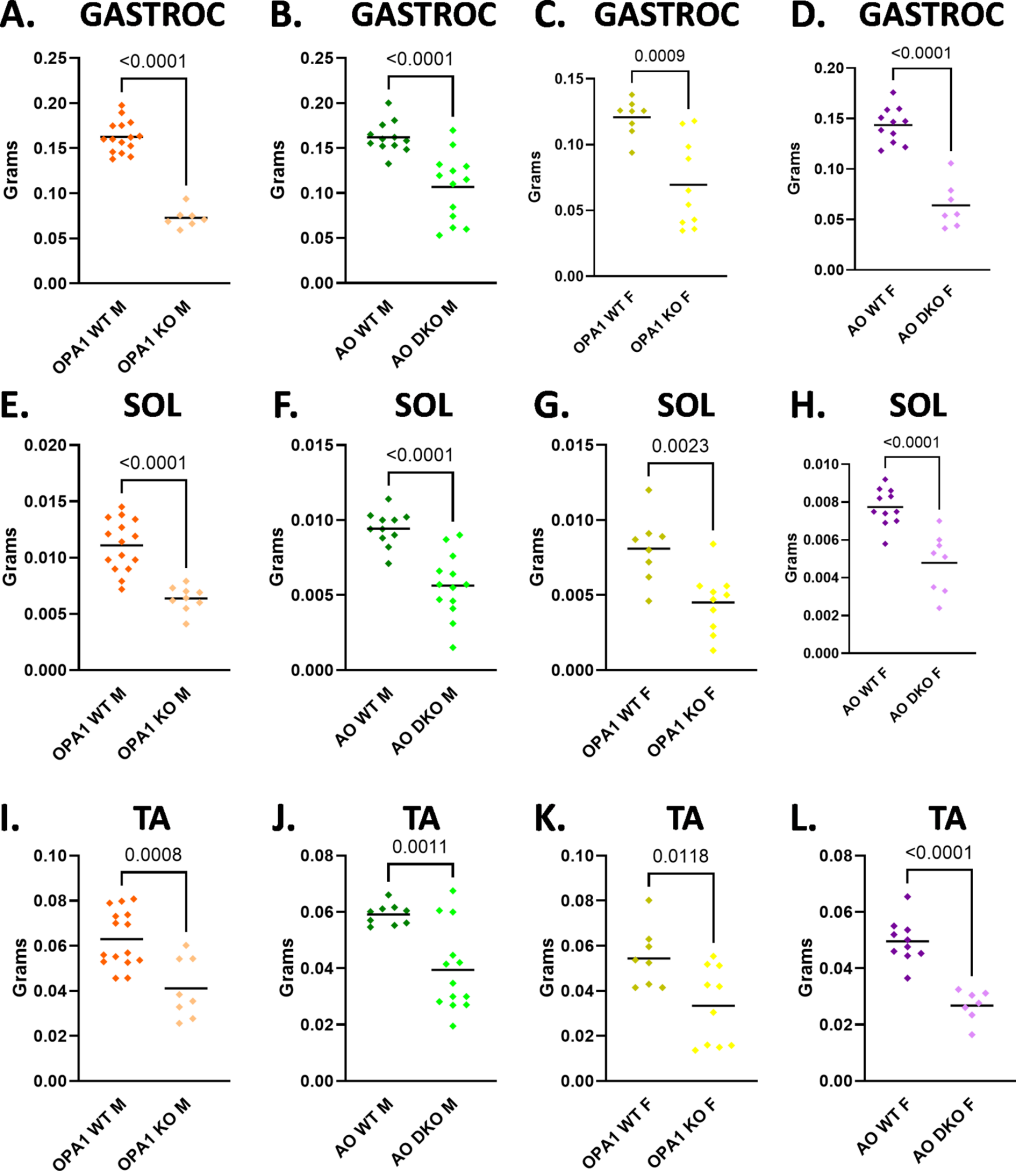
Loss of muscle mass in mOPA1 and mAO DKO mice. mOPAI KO males have a loss in lean muscle mass in multiple muscle groups including gastrocnemius (GASTROC), soleus (SOL), and tibialis anterior (TA) **(A, E, I)**. mOPA1 females have decreased muscle mass in all muscle groups **(C, G, K)**. MAO DKO males and females have significant loss of muscle mass compared to WT **(B, D, F, H, J, L)**. Created using BioRender.com.

Depletion of OPA1 in skeletal muscle induces muscle atrophy (9). ATF4 is a known mediator of skeletal muscle atrophy (30). We hypothesized that ATF4 is necessary for skeletal muscle atrophy in mOPA1 KO mice and sought to determine if loss of ATF4 would reverse muscle atrophy in this model. We confirmed that mOPA1 males exhibit loss of lean muscle mass in multiple muscle groups including gastrocnemius, soleus, and tibialis anterior (**Figures 4A, E, I**). Likewise, mOPA1 females also have decreased muscle mass in these muscle groups (**Figures 4C, G, K**). Similarly, mAO DKO males and females also had a significant loss of muscle mass compared to WT, indicating that ATF4 does not mediate muscle atrophy in response to loss of OPA1 in skeletal muscle (**Figures 4B, D, F, H, J, L**). The degree of muscle mass loss for all skeletal muscle groups was approximately 40%, and there was no difference in magnitude of muscle mass loss between mOPA1 KOs and mAO DKOs, with the exception of there being less gastrocnemius muscle loss in mAO DKO males compared to mOPA1 KO males.

### ATF4 deletion does not reverse metabolic adaptations arising from OPA1 skeletal muscle deficiency

**Figure 5:**
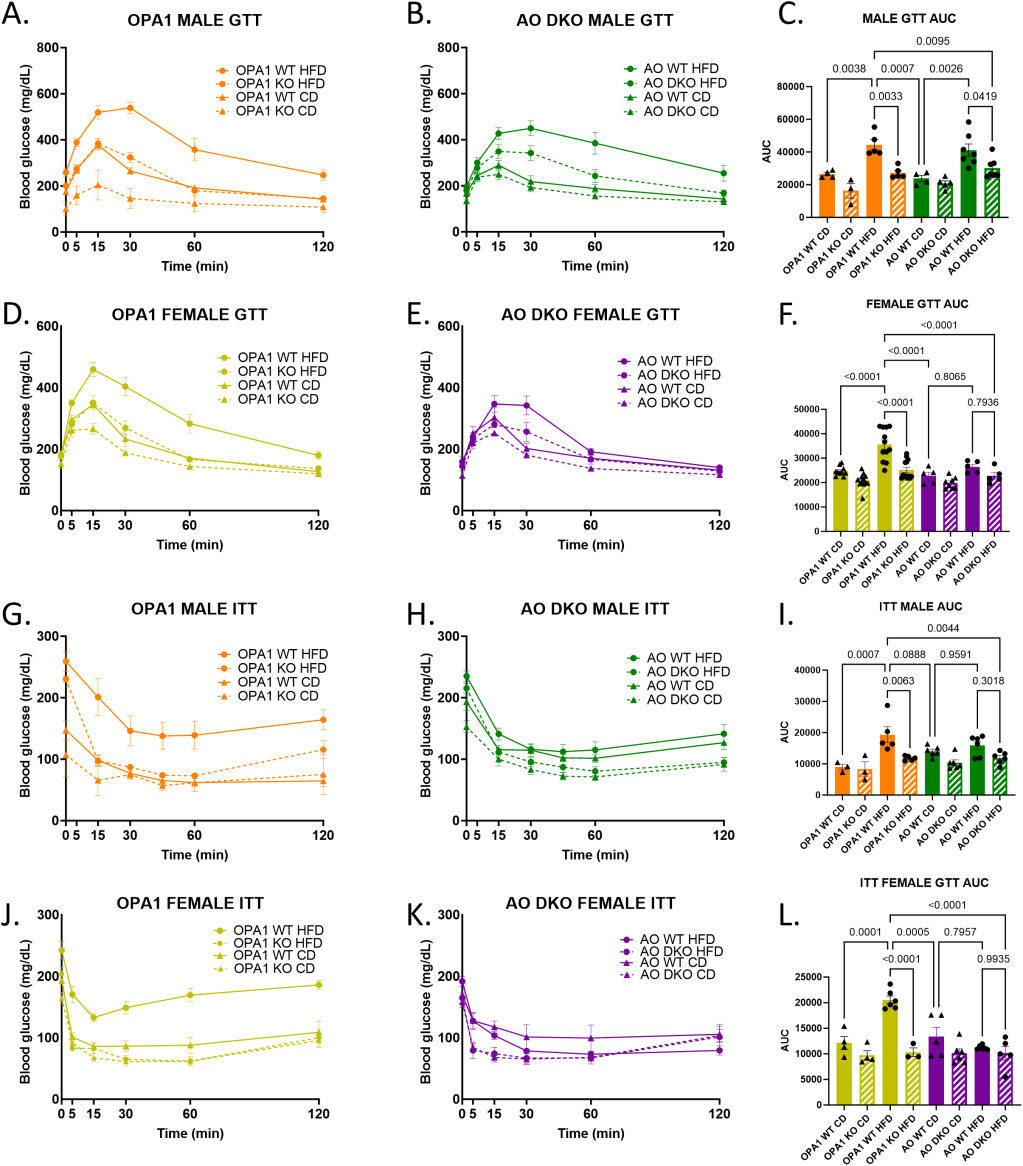
Glucose Tolerance Testing (GTT) and Insulin Tolerance Testing (ITT). GTT and Area under the curve (AUC) for males (**A-C**, n=3-7), and females (**D-F**, n=5-12), male ITT (**G-I**, n=3-6), and female ITT (**J-L**, n=3-6). Created using BioRender.com.

mOPA1 KO mice have improved glucose tolerance and maintain their insulin sensitivity even when subjected to HFD. We hypothesized that if ATF4 was the major regulator of FGF21 release from skeletal muscle, then loss of ATF4 in this model would abrogate metabolic protection leading to glucose intolerance and insulin resistance. As previously observed, mOPA1 KO male mice on HFD have improved glucose tolerance compared to WT on HFD (**Figures 5A, C**). Likewise, mOPA1 KO female mice on HFD have improved glucose tolerance compared to WT on HFD (**Figures 5D, F**). Like mOPA1 KO males, mAO DKO males on HFD have improved glucose tolerance compared to mOPA1 WT males on HFD (**Figures 5B, C**). mOPA1 KO females on HFD exhibited improved glucose tolerance relative to mOPA1WT on HFD. The degree of glucose intolerance following HFD in mAO WT was not as striking as observed in the mOPA1 WT cohorts. There was a marginal difference in glucose tolerance relative to mAO WT and a significant reduction relative to mOPA1 WT (**Figures 5E, F**). Regarding insulin sensitivity, mOPA1 KO males and females on HFD have improved insulin sensitivity compared to mOPA1 WT on HFD (**Figures 5G, J**). A similar pattern was observed in mAO DKO male and female mice. mAO WT on HFD did not develop the same degree of insulin resistance as mOPA1KO WT mice and statistical differences in insulin tolerance was not observed when HFD AO WT and AO DKO were compared (**Figures 5H, I, K, L**).

### Sex dimorphism in expression and circulating levels of FGF21 and GDF15

**Figure 6:**
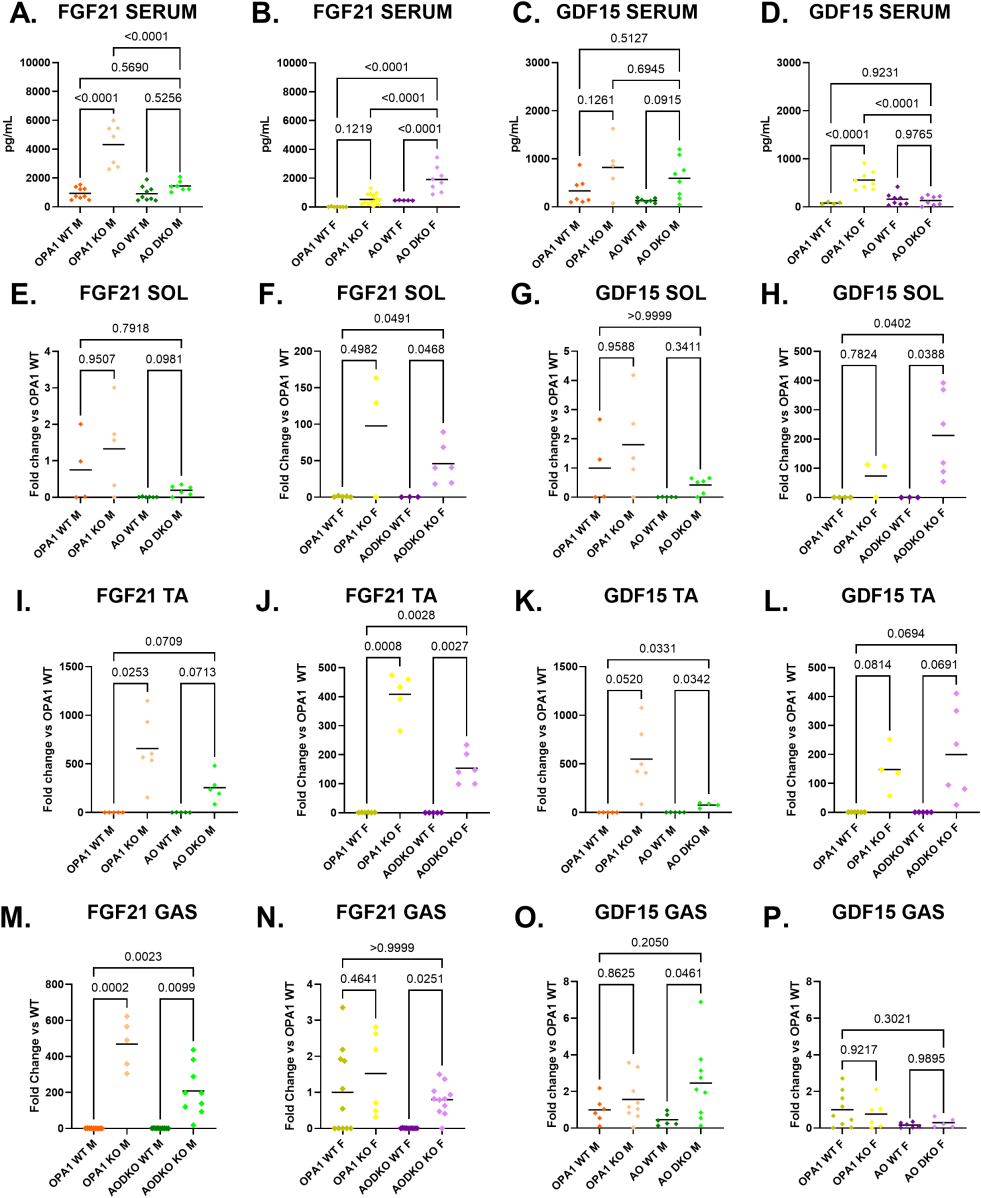
FGF21 and GDF15 levels in serum and skeletal muscle. Serum FGF21 (**A** males n=8-12, **B** females n=5- 9). Serum GDF15 (**C** males n-5-8, **D** females n-4-8). Tissue source of FGF21 and GDF15 assessed by measurement of mRNA induction in skeletal muscle (n=4-11): soleus (SOL, **E-H**), tibialis anterior (TA, **I-L**), and gastrocnemius (GAS, **M-P**). Created using BioRender.com.

As previously described, skeletal muscle mRNA expression and circulating levels of FGF21 were increased in mOPA1 KO males (**Figure 6A**). Deletion of ATF4 attenuated FGF21 mRNA and significantly lowered circulating concentrations of FGF21 in mAO DKO males (**Figure 6A**). In mOPA1 KO females, increased circulating concentrations of FGF21 was not observed (**Figure 6B**). Unexpectedly, deletion of ATF4 was associated with induction of FGF21 mRNA and increased circulating concentrations of FGF21 in mAO DKO females (**Figure 6B**).

Since mAO DKO males and mOPA1 female maintain resistance to DIO despite lack of induction of FGF21, we sought to determine alternative circulating factors that could account for the lean phenotype in these two groups. We investigated circulating levels of GDF15, which has been shown to be induced as a mitokine and to mediate metabolic adaptations and resistance to DIO. GDF15 serum concentration did not change in mOPA1 KO males compared to WT (**Figure 6C**). There was a trend for increased GDF15 in mAO DKO males, but not statistically significant (**Figure 6C**, p=0.0915). GDF15 serum level was significantly increased in mOPA1 females, but not mAO DKO females compared to WT (**Figure 6D**).

**Figure 7:**
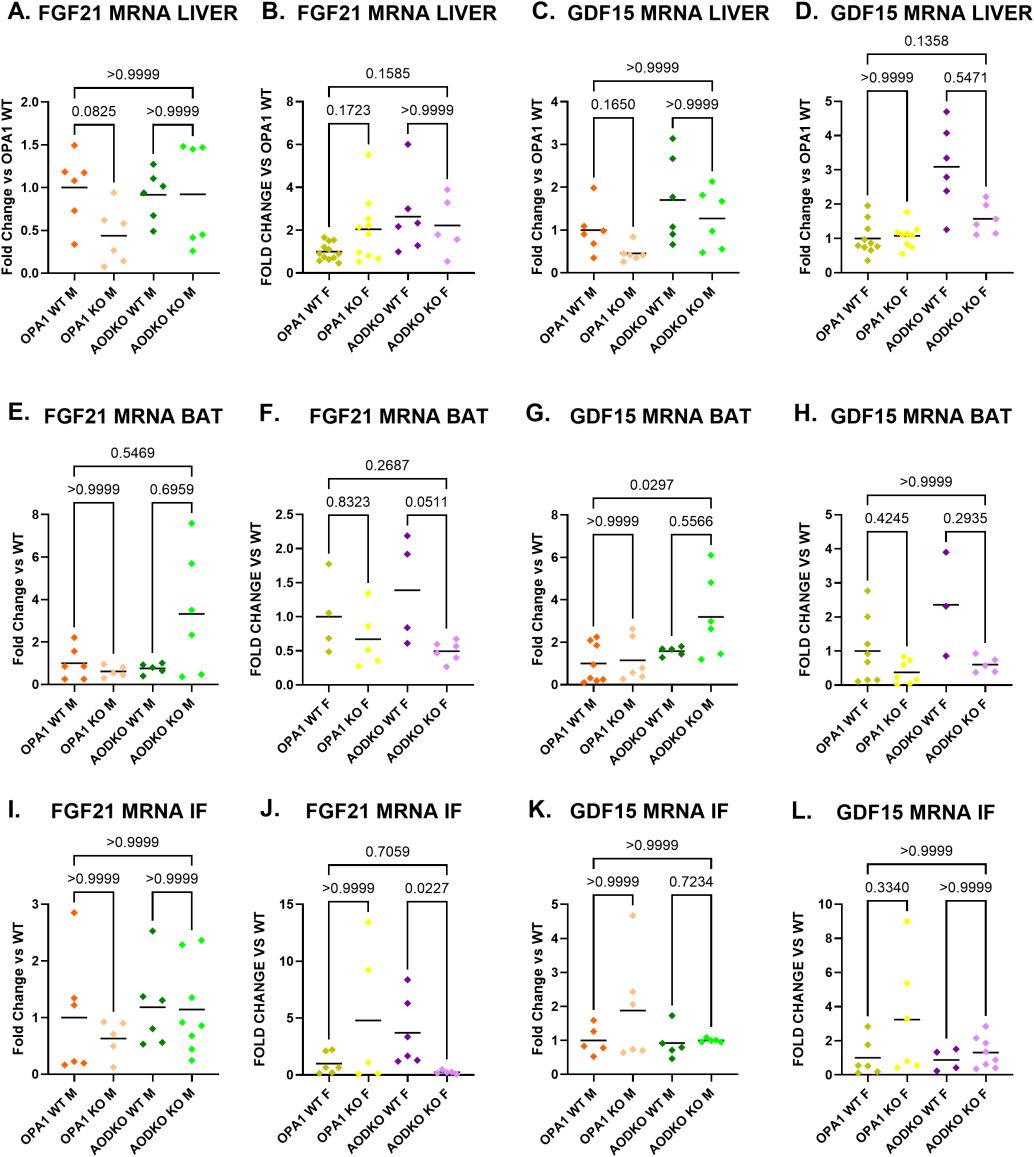
FGF21 and GDF15 levels in liver and fat. Tissue source of FGF21 and GDF15 assessed by measurement of mRNA induction in liver (**A-D**, n=6-10), brown adipose tissue (BAT, **E-H**, n=4-8), and inguinal fat (IF, **I-L**, n-5-8). Created using BioRender.com.

To determine the tissue source of the circulating FGF21 and GDF15, we measured mRNA induction in skeletal muscle (soleus, tibialis anterior, and gastrocnemius), liver, brown adipose tissue, and inguinal fat (**Figures 6E–P**, **7A–L**). In mOPA1 males, increased serum FGF21 was associated with increased expression of *Fgf21* in tibialis anterior and gastrocnemius (**Figures 6A, I, M**). In mAO DKO females, increased serum FGF21 was associated with increased expression of *Fgf21* in soleus, tibialis anterior, and gastrocnemius (**Figures 6B, F, J, N**). In mAO DKO males, the trend for increased GDF15 was associated with increased *Gdf15* expression in tibialis anterior and gastrocnemius (**Figures 6C, K, O**). In mOPA1 females, the increased serum GDF15 was associated with a trend for increased *Gdf15* expression in tibialis anterior, which did not reach statistical significance (**Figures 6D, L**). There were no significant changes in expression levels of *Fgf21* or *Gdf15* in liver nor white or brown adipose tissue of mOPA1 KO or mAO DKO mice of any sex compared to WT (**Figures 7A–L**).

## Discussion

A growing body of literature has revealed a connection between mitochondrial stress in skeletal muscle and favorable systemic metabolic phenotypes (9, 31–38). Our previous study showed that depletion of OPA1 in skeletal muscle (mOPA1 KO) in male mice induces mitochondrial stress, induction of the ER stress and ISR, including increased expression of ATF4, increased expression of FGF21, and FGF21-dependent protection from HFD-induced diabetes and obesity (9). FGF21 is a known regulator of whole-body metabolism and is expressed in liver, pancreas, brain, white adipose tissue, brown adipose tissue, and skeletal muscle (3, 4, 9, 11, 13, 18, 33, 39–41). Therefore, understanding the tissue-specific cell signaling pathways, transcription factors, and systemic signaling molecules responsible for FGF21 expression leading to these favorable phenotypes could yield useful therapeutic targets for the treatment of diabetes and obesity. Although ATF4 is known to be critical for *Fgf21* expression in various models and tissues, *Fgf21* can be regulated by multiple transcription factors (3, 4, 13, 25). This study sought to determine if ATF4 is necessary for *Fgf21* induction or the metabolically favorable phenotype in the mOPA1 KO model. Since ChIP-seq in *ex vivo* skeletal muscle cells poses many technical challenges, we analyzed direct ATF4:FGF21 interactions under conditions mimicking the ER stress induction observed in mOPA1 KOs using GEO DataSet analysis in WT and *Atf4* KO MEFs treated with the ER-stress-inducer tunicamycin (17). RNA-seq of these cells revealed that FGF21 is expressed in WT but not in ATF4 KO cells, indicating that ATF4 is necessary and sufficient for FGF21 induction in these cells in response to ER stress. Direct ATF4 DNA binding was demonstrated by the GEO DataSet ChIP-seq analysis, indicating that ATF4 binds directly to the promoter of *Fgf21* in WT MEFS, but not in *Atf4* KO MEFs following tunicamycin treatment. Although prior studies have shown ATF4 binding to FGF21 in response to autophagy inhibition in MEFs (33) and Angiotensin II stimulation in human aortic vascular smooth muscle cells (14), this is the first study to demonstrate ATF4 binding to the FGF21 promoter in response to ER stress. However, this analysis does not preclude the possibility that other transcription factors could also regulate FGF21 expression in the mOPA1 KO model of ER stress in skeletal muscle.

Creation of the mAO DKO mouse enabled us to investigate the contribution of ATF4 to multiple outcomes seen in the mOPA1 KO mice including activation of the ISR, FGF21 induction, glucose homeostasis, insulin sensitivity and protection from diet-induced obesity. Pereira et al. reported that mOPA1 male mice have induction of multiple ISR markers including ATF4, XBP1, and CHOP in skeletal muscle (9). In this study, we find that ATF6, XBP1 and CHOP are induced in mAO DKO males compared to WT, suggesting that the induction of these ER stress markers is independent of ATF4.

Unlike mOPA1 KO and mAO DKO males, we report here that mOPA1 KO and mAO DKO females do not have induction of ATF4, XBP1s, ATF6, BiP, or CHOP. This is consistent with previously reported sex-dependent differences in ER stress response in other models including tunicamycin-induced acute kidney injury in mice, which revealed induction of BiP, XBP1s, and CHOP was greater in males than females and mediated via testosterone (42). Additional studies have investigated sex-dependent differences in ISR markers in liver and found increased XBP1s in males following growth hormone administration and higher levels of BiP, ATF4, and XBP1 in prepubertal males following testosterone administration (43, 44). Furthermore, a recent study found that there is a difference in diurnal expression of ISR factors Atf4 and Chop in males and females (45). Since all serum and skeletal muscle samples were collected between 8 am and noon for our study, we cannot exclude the possibility that female peak expression of ISR factors occurred outside of that timeframe and was not captured. The absence of ATF4 induction in female mOPA1 KO and the increase in circulating FGF21 and expression in multiple muscle groups in mAO DKO females further supports that FGF21 release from skeletal muscles of female mice is ATF4-independent. These studies add to a growing body of literature demonstrating sex-dependent differences in ISR markers and may point to these differences as influencing sex-dependent disease outcomes.

As previously reported, mOPA1 KO males are protected from diet-induced obesity (DIO) on HFD compared to WT males (9). We report here that mOPA1 KO females are also protected from DIO compared to WT females. We hypothesized that ATF4 induction, as a key regulator of FGF21 expression would be required for this lean phenotype. If our hypothesis were correct, then mAO DKO mice would not be protected from DIO. Contrary to our expectation, mAO DKO males and females remain resistant to DIO on HFD relative to WTs. Overall, body fat mass and body fat percent are decreased in both mOPA1 KO and mAO DKO males and females compared to their WT counterparts, suggesting that ATF4 was not the sole regulator of the metabolic adaptations to OPA1 deficiency. This is consistent with a similar mouse model, skeletal muscle-specific deletion of mitoprotease LONP1, which is resistant to diet-induced obesity independently of ATF4 (31). These observations also raise the possibility that additional ISR outputs might compensate for the absence of ATF4 in mediating systemic adaptations to OPA1 deficiency in skeletal muscle. The phenotypic similarity between male and females, despite the absence of induction of ISR outputs in females indicate overlapping and redundant regulation of these pathways in skeletal muscle.

Since depletion of OPA1 in skeletal muscle is known to cause muscle atrophy, and expression of ATF4 in skeletal muscle is an established mediator of skeletal muscle atrophy, we hypothesized that ATF4 may mediate skeletal muscle loss, in mOPA1 KO mice, such that muscle loss would be mitigated in mAO DKO mice (30). As previously demonstrated, OPA1 males have reduced lean muscle mass in multiple muscle groups including gastrocnemius, soleus, and tibialis anterior. Likewise, mOPA1 females also have decreased muscle mass in these muscle groups. Surprisingly, mAO DKO males and females continued to exhibit reduced muscle mass that was similar to mOPA1 males and females. Although prior studies revealed that OPA1 deficiency leads to increased oxidative stress, which activates the ER stress response, including ATF4 upregulation, leading to muscle atrophy (10), our study indicates that muscle atrophy in mOPA1 KO mice is mediated by a mechanism largely independent of ATF4, potentially involving alternative transcription factors that are induced following ISR activation. This notion is supported by literature that links ISR transcription factors, such as CHOP and XBP1 to muscle atrophy (46). Since these ISR markers are not upregulated in mOPA1 KO or mAO DKO females, there remain unidentified factors responsible for the persistent muscle atrophy in these mice.

mOPA1 KO mice exhibit preserved glucose homeostasis and remain insulin sensitive following HFD. If ATF-mediated *Fgf21* induction is necessary for these metabolic phenotypes, then loss of ATF4 in this model would have been predicted to impair glucose tolerance and promote insulin resistance. Since mAO DKO males on HFD have improved glucose tolerance compared to mAO WT males on HFD and insulin sensitivity compared to OPA1 WT males on HFD, it suggests that ATF4 is not the sole mediator of the metabolic adaptations observed in mOPA1 KO males. Prior studies have suggested that alternative ISR factors such as CHOP can regulate glucose tolerance and insulin resistance, which may account for the metabolic phenotype in the mAO DKO males (47, 48). mAO DKO females on HFD do not have improved glucose tolerance or insulin sensitivity relative to mAO WT females on HFD. This may be due in part to reduced development of glucose intolerance and insulin resistance in mAO WT females on HFD. Prior studies have demonstrated reduced risk of females developing glucose intolerance or diabetes compared to males. ORMDL3TG male mice demonstrate greater impaired glucose homeostasis, insulin resistance, increased induction of the UPR/ER stress and development of non-alcoholic steatohepatitis (NASH) compared to females on HFD (49). Additionally, premenopausal women have a lower risk of developing diabetes than men and postmenopausal women. The apparent protective effect of estrogen may explain why mAO WT females did not develop impaired glucose tolerance. However, it remains unclear why mOPA1 WT females developed diet-induced glucose intolerance although mAO WT females did not. All mice were on the same genetic background and utilized the same floxed alleles and Cre promoter. Differences between these groups may be attributable to the lower baseline expression of ISR markers ATF6 (p=0.0337) and CHOP (p=0.0090) in mAO WT females compared to mOPA1 WT females, as well as additional regulators of glucose homeostasis and insulin resistance that were not assessed in this study.

In this study, we found that FGF21 serum levels are increased by >4-fold in mOPA1 males and mAO DKO females, but FGF21 serum levels did not significantly increase in mAO DKO males or mOPA1 females, indicating ATF4 expression partially regulated serum FGF21 levels in these models. However, the absence of complete suppression, suggest that other transcriptional mechanisms could be at play. Prior studies have implicated multiple ISR factors in the regulation of FGF21 including CHOP-mediated control of FGF21 expression and secretion in a thapsigargin-induced ER stress model and XBP1s-mediated activation of FGF21 in liver following intraperitoneal administration of tunicamycin in mice (17, 25). There are numerous known transcription factors that regulate FGF21 (50), and since neither CHOP nor XBP1s are induced in mAO DKO females, the transcription factor necessary for FGF21 induction in this model remains to be identified. Additionally, due to the sexual dimorphism in FGF21 induction in our models, our findings add to the limited existing literature demonstrating sex-dependent difference in FGF21 secretion in response to various stimuli (45, 51–53). In contrast, there are numerous studies investigating FGF21 expression solely in male or female subjects, but no direct comparison between sexes (54–58). This highlights the need for future animal and human studies to include both males and females to delineate sex-specific differences in FGF21 regulation. Surprisingly, this study showed that mAO DKO males and mOPA1 females maintain resistance to DIO despite attenuation of induction of FGF21, even though FGF21 is necessary for the lean phenotype in mOPA1 FGF21 DKO male mice (9). However, our study is consistent with previous findings that FGF21 is dispensable for metabolic improvements evoked by compromised mitochondrial function in skeletal muscle (59). Since FGF21 was not the sole mediator of the observed metabolic phenotype, we also tested the possibility that additional mitokines could contribute to the lean phenotype in addition to, or instead of FGF21. GDF15 is a mitokine that acts in a manner similar to FGF21, that may increase energy expenditure and protect against DIO (60, 61). Additionally, GDF15 has been demonstrated to be increased following ISR induction in multiple models of mitochondrial stress (61–63). In this study, we found that serum GDF15 levels are induced in a pattern that appear to be inverse to FGF21 serum levels. Specifically, while FGF21 was clearly induced in mOPA1 KO males, GDF15 was not. However, in mAO DKO males, FGF21 was not induced, but there was a trend of induction of GDF15. A parallel but inverse phenomenon was observed in females. GDF15 was induced in mOPA1 KO females, while FGF21 was not increased. Conversely, GDF15 was not induced in mAO DKO females, while FGF21 was significantly induced. Thus, GDF15 could play a compensatory role in controlling energy homeostasis in the absence of significant FGF21 induction. Indeed a role for GDF15 was recently implicated in a model of brown adipose tissue mitochondrial stress induced by OPA1 deficiency (61). GDF15 has been reported to be regulated by multiple ISR transcription factors including CHOP, GRP78 (glucose-regulated protein 78), and ATF3 (Activating Transcription Factor 3) (64–66). These studies support that alternative ISR transcription factors may be regulating GDF15 in the absence of ATF4 in our models.

Since *Fgf21* and *Gdf15* (67–69) can be induced in various tissues, our study also sought to determine the tissue source of circulating FGF21 and GDF15. Since our Cre promoter is for skeletal muscle, we predicted that *Fgf21* and *Gdf15* mRNA induction would occur in skeletal muscle (soleus, tibialis anterior, and gastrocnemius), but not in alternative tissues that are known to produce circulating FGF21 and GDF15: liver, brown adipose tissue, and inguinal fat. We found there were no significant changes in expression levels of *Fgf21* or *Gdf15* in liver nor white or brown adipose tissue of mOPA1 KO or mAO DKO mice of any sex compared to WT.

Since serum FGF21 is increased in mOPA1 males, we anticipated that it would be increased in all skeletal muscle groups tested. It was induced in tibialis anterior and gastrocnemius, but unexpectedly not soleus. Since AO DKO males did not have an increase in serum FGF21, we predicted we would not see an increase in skeletal muscle expression of *Fgf21*. Surprisingly, we observed induction of *Fgf21* in AO DKO male gastrocnemius, and a trend for induction in soleus and tibialis anterior. Similarly, mOPA1 KO females did not have an increase in serum FGF21 but did have an increase in expression of FGF21 in tibialis anterior. This may suggest that either FGF21 is not being secreted from these skeletal muscles, or that a certain threshold of FGF21 release needs to occur in skeletal muscle before it can be detected in circulation. In mAO DKO females, increased serum FGF21 is associated with increased expression of *Fgf21* in soleus, tibialis anterior, and gastrocnemius. While prior groups have assessed FGF21 expression in combined muscle groups (quadriceps and gastrocnemius (36) or gastrocnemius and tibialis anterior (70)), this study is the first to directly compare induction of FGF21 in distinct muscle groups and find that FGF21 is not induced to the same degree in all muscles and induction in one or more skeletal muscle groups is not always associated with increased circulating FGF21.

In similar fashion GDF15 serum level did not perfectly correlate with tissue expression. In mOPA1 KO females, GDF15 serum level was significantly increased, but there was only a trend for increased expression of *Gdf15* in tibialis anterior, and no increase in gastrocnemius or soleus. We did not assess all skeletal muscle groups, so it is possible that serum GDF15 in mOPA1 females is coming from a different muscle group. In mAO DKO males, the trend for increased GDF15 is associated with increased *Gdf15* expression in tibialis anterior and gastrocnemius. These findings of differential expression of *Gdf15* in individual muscle groups is consistent with a prior study investigating the effect of mitochondrial stress in skeletal muscle (induced by UCP1 (uncoupling protein 1) transgenic over-expression in skeletal muscle) on *Gdf15* expression (35). This study found that in the setting of mitochondrial stress, *Gdf15* expression is differentially expressed in extensor digitorum longus, tibialis anterior, gastrocnemius, quadriceps, and soleus and that increased skeletal muscle expression of *Gdf15* is associated with increased serum concentration of GDF15. Additionally, similar to our study, the UCP1 study utilized male and female mice and found that serum GDF15 is increased in both sexes. Furthermore, since a recent study found that there is an approximate 4-hour delay between peak mRNA expression in skeletal muscle and peak serum levels of FGF21 and GDF15, we cannot exclude the possibility that the imperfect correlation we see between skeletal muscle expression and serum concentration are due to timing of collection (45).

Our study determined that ATF4 is not the sole mediator responsible for increasing circulating FGF21 or GDF15. We also identified GDF15 as a potential candidate that may mediate the metabolic adaptations, particularly under conditions in which FGF21 induction is attenuated. This is consistent with findings by Ost et al. that revealed mitochondrial stress promotes GDF15 secretion from skeletal muscle in mice, promotes insulin sensitivity, and attenuates weight gain, and these metabolic outcomes are reversed with *Gdf15* KO in skeletal muscle (35). Our working model is that skeletal muscle-specific deletion of OPA1 causes activation of the ISR including upregulation of ER stress transcription factors in a genotype- and sex-dependent manner, which induce FGF21 and GDF15 expression via ATF4 dependent and independent pathways, leading to protection from diabetes and obesity. Although multiple models have demonstrated that increases in circulating FGF21 correlates with metabolically favorable phenotypes, including resistance to the metabolic syndrome that develops in response to HFD, studies that have attempted to use FGF21 analogs for the treatment of metabolic syndrome in humans have had limited success (28, 29). The failure may be due, in part, to species differences in FGF21 modulation of systemic metabolism or the requirements for additional circulating mitokines, such as GDF15, to achieve full metabolic protection. It is possible that deletion of *Opa1* in skeletal muscle induces the ISR to generate a specific translational program that results in the expression and secretion of multiple unique mitokines including FGF21 and GDF15, that are necessary for the favorable metabolic phenotype in these models. Future studies will characterize the roles of CHOP, XBP1s, and GDF15 and the full transcriptional program necessary for protection from HFD-induced obesity and diabetes in mOPA1 mice, and the mechanistic basis for sex differences in their regulation.

## Data Availability

The original contributions presented in the study are included in the article/**Supplementary Material**, further inquiries can be directed to the corresponding author.
