# Supplementary figures and images for: ATF4-dependent and independent mitokine secretion from OPA1 deficient skeletal muscle in mice is sexually dimorphic
Source: Front Endocrinol (Lausanne). 2024 Sep 24;15:1325286. doi: 10.3389/fendo.2024.1325286 (PMC11458430; doi:10.3389/fendo.2024.1325286)

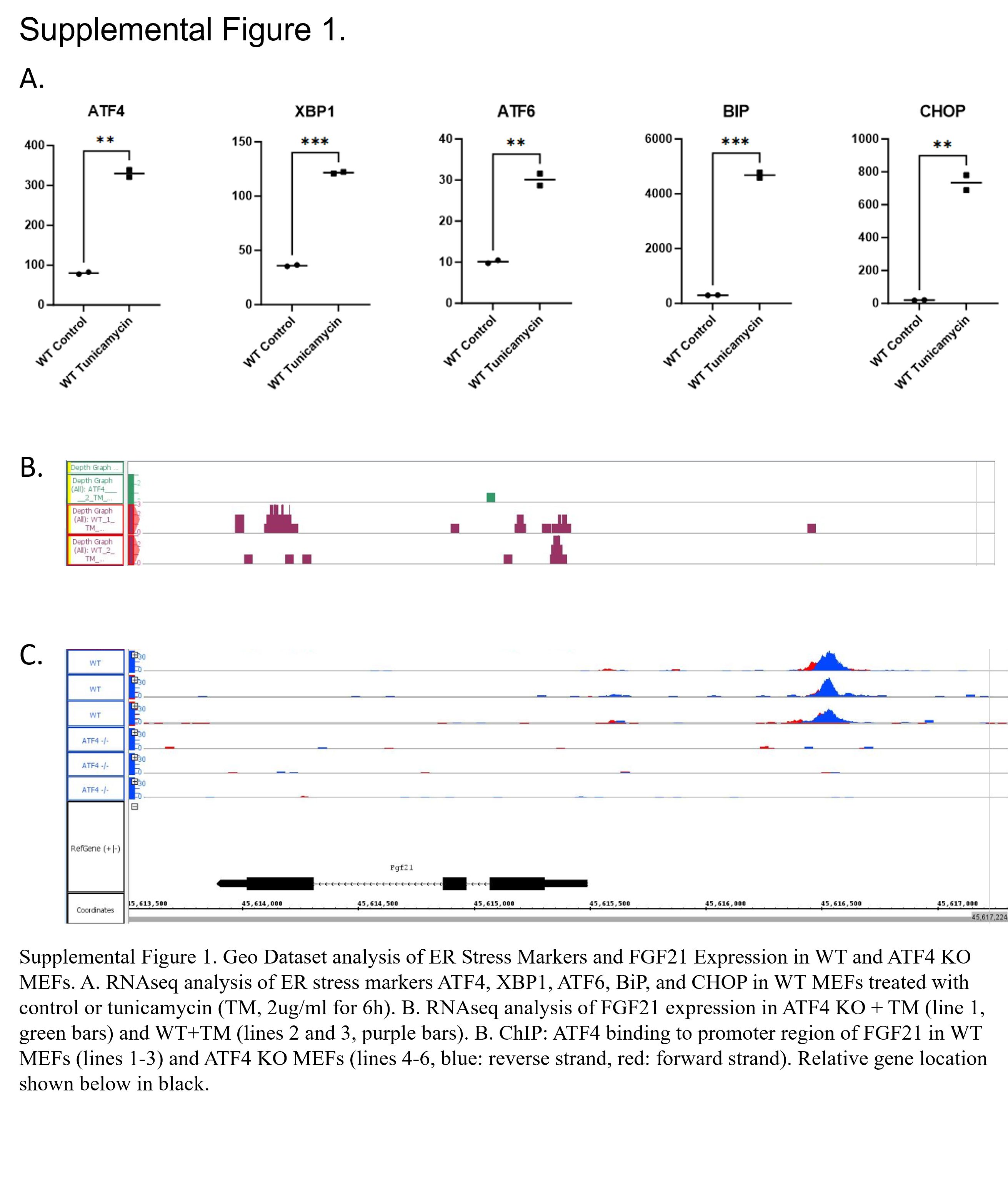

Supplement: Supplementary file 1 [file Image1.tif]

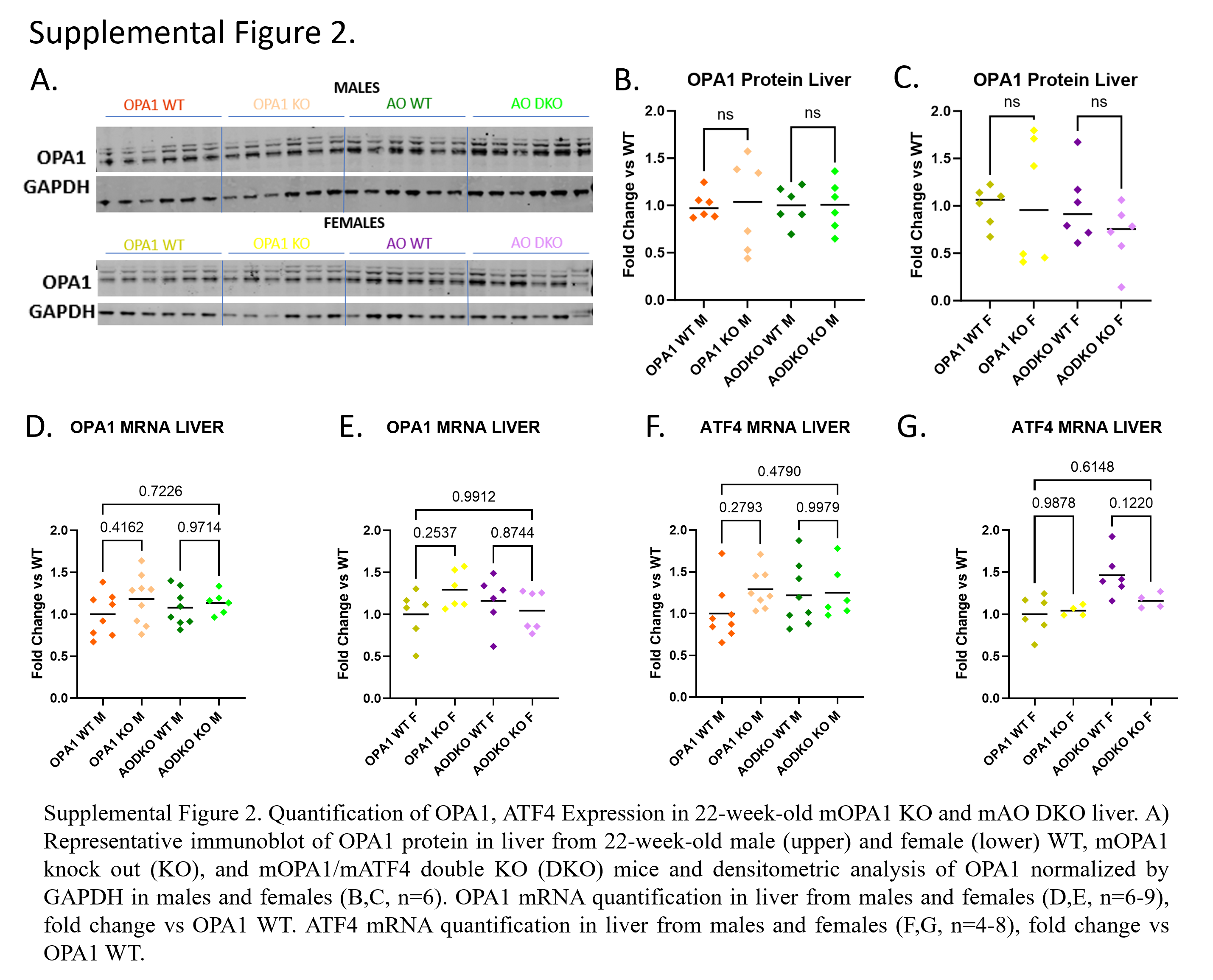

Supplement: Supplementary file 2 [file Image2.tif]

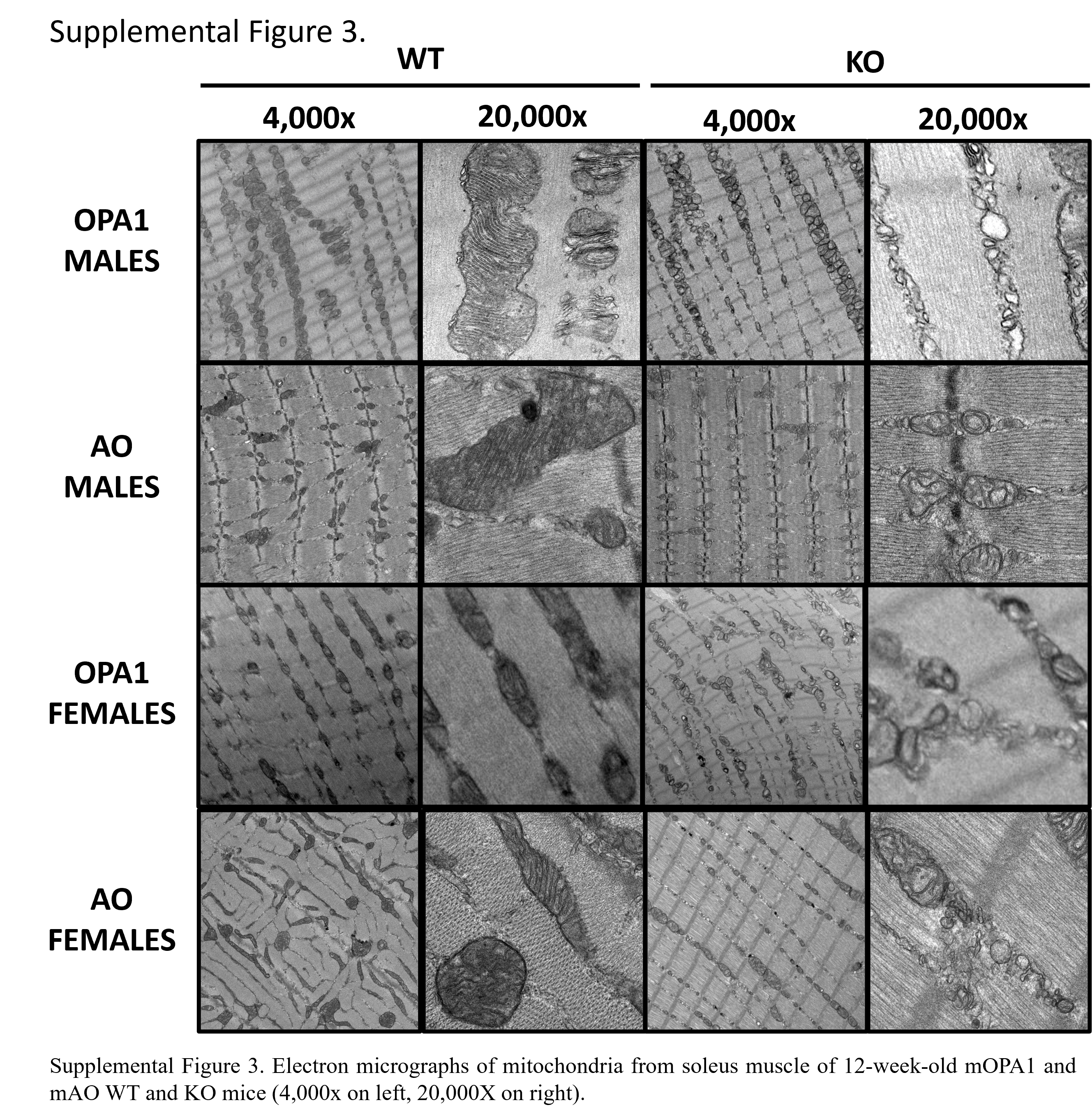

Supplement: Supplementary file 3 [file Image3.tif]
